# Determinants of Inappropriate Antibiotics Use in Rural Central Ghana Using a Mixed Methods Approach

**DOI:** 10.3389/fpubh.2020.00090

**Published:** 2020-03-24

**Authors:** Samuel Afari-Asiedu, Felix Boakye Oppong, Alma Tostmann, Martha Ali Abdulai, Ellen Boamah-Kaali, Stephaney Gyaase, Oscar Agyei, John Kinsman, Marlies Hulscher, Heiman F. L. Wertheim, Kwaku Poku Asante

**Affiliations:** ^1^Kintampo Health Research Centre, Ghana Health Service, Kintampo, Ghana; ^2^Radboud University Medical Center, Institute for Health Sciences, Nijmegen, Netherlands; ^3^Department of Medical Microbiology and Radboudumc Center for Infectious Diseases, Radboudumc, Nijmegen, Netherlands; ^4^Department of Epidemiology and Global Health, Umeå University, Umeå, Sweden

**Keywords:** antibiotics, antibiotic use, inappropriate antibiotic use, antibiotic resistance, Ghana

## Abstract

**Background:** The consequences of antibiotic resistance are projected to be most severe in low and middle income countries with high infectious disease burden. This study examined determinants of inappropriate antibiotic use at the community level in rural Ghana.

**Methods:** An observational study involving qualitative and quantitative methods was conducted between July, 2016 and September, 2018 in Ghana. Two household surveys were conducted at two time points (2017 and 2018) among 1,100 randomly selected households over 1 year. The surveys focused on antibiotic use episodes in the past month. Four in-depth interviews and two focus group discussions were performed to further explain the survey results. Determinants of inappropriate antibiotic use were assessed using a mixed effect logistic regression analysis (multilevel analysis) to account for the clustered nature of data. We defined inappropriate antibiotic use as either use without prescription, not completing treatment course or non-adherence to instruction for use. Qualitative data were thematically analyzed.

**Results:** A total of 1,100 households was enrolled in which antibiotics were used in 585 (53.2%) households in the month prior to the surveys. A total of 676 (21.2%) participants out of 3,193 members from the 585 reportedly used antibiotics for 761 episodes of illness. Out of the 761 antibiotic use episodes, 659 (86.6%) were used inappropriately. Paying for healthcare without health insurance (Odds Ratio (OR): 2.10, 95% CI: 1.1–7.4, *p*-value: 0.026), not seeking healthcare from health centers (OR: 2.4, 95% CI: 1.2–5.0, *p*-value: 0.018), or pharmacies (OR: 4.6, 95% CI: 1.7–13.0, *p*-value: 0.003) were significantly associated with inappropriate antibiotic use. Socio-demographic characteristics were not significantly associated with inappropriate antibiotic use. However, the qualitative study described the influence of cost of medicines on inappropriate antibiotic use. It also revealed that antibiotic users with low socioeconomic status purchased antibiotics in installments which, could facilitate inappropriate use.

**Conclusion:** Inappropriate antibiotic use was high and influenced by out-of-pocket payment for healthcare, seeking healthcare outside health centers, pharmacies, and buying antibiotics in installments due to cost. To improve appropriate antibiotic use, there is the need for ministry of health and healthcare agencies in Ghana to enhance healthcare access and healthcare insurance, and to provide affordable antibiotics.

## Background

The global increase in antibiotic resistance is threatening and potentially reversing the advances made against infectious diseases (1, 2). It is estimated that antimicrobial resistance (AMR) including antibiotic resistance could cause 10 million deaths a year by 2050 if the appropriate steps are not taken soon (3). The consequences of these are projected to be most severe in low and middle income countries (LMIC), especially Africa where infectious disease burden is high and alternative antibiotics are often unavailable or costly (4).

Antibiotic-resistant bacteria have historically been hospital-based, but they are now becoming more common in the community (5, 6). In LMIC inappropriate antibiotic use is an important driver of resistance, and it is common as antibiotics are bought or obtained without prescription from private, including illegal providers (7, 8). Patients who purchase antibiotics from these sources have the flexibility to buy in smaller quantities if they cannot afford a full course, which contributes to inappropriate use (9). Inappropriate antibiotic use encompasses use that are not in accordance with international treatment guidelines, including but not limited to: taking antibiotics without prescription, not completing the course, taking an insufficient dose, taking antibiotics for wrong indications and sharing antibiotics (10). A patient's decision to seek healthcare or use antibiotics is influenced by contextual factors, like: access and distance to appropriate health care provider, income and the general economic circumstances of users and the cultural norms that have developed around medicine use over a period of time (7). In Ghana, the National Health Insurance Scheme (NHIS) also influences healthcare accessibility. The NHIS provides registered members with healthcare for some common diseases such as malaria, upper respiratory tract infections and diarrheal without paying cash where applicable (11).

A recent systemic review in sub-Saharan Africa revealed a high level of resistance to commonly used antibiotics. Specifically, 90% of Gram-negatives were resistant to chloramphenicol, a commonly used antibiotic (12). In Ghana, studies have shown high resistance to commonly used antibiotics such as tetracycline, ampicillin, chloramphenicol and cotrimoxazole (13, 14). Penicillin is rapidly losing its effectiveness against *Streptococcus pneumoniae* and *Neisseria meningitidis* in Ghana, partly driven by inappropriate antibiotic use (15, 16). However, a gap exists in the understanding of what actually drives antibiotic use (9), particularly within households at the community level. Consequently interventions aimed at improving antibiotic access and use are constrained by the paucity of information on determinants of antibiotic use (17). Gaining insight into these factors will contribute to the development of more context-specific and effective policies and interventions to improve antibiotic use and curb resistance.

This paper examines inappropriate antibiotic use and the household factors which influence use at the community level in rural Ghana.

## Methods

### Study Design

An observational study was conducted using quantitative and qualitative methods to examine household factors which influence antibiotic use among community members in Ghana between January 2016 and July, 2018 (18). The study employed three consecutive approaches. First, a qualitative approach was taken to explore the factors which affect community antibiotic access using in-depth interviews (IDIs) and focus group discussions (FGDs) as reported in a previous paper (19). Second, the results of the qualitative study were used to refine a questionnaire which was deployed at the household level. A total of 1,100 households was contacted at two time points (six month apart) to capture antibiotic use over 1year. Third, in order to deepen our understanding of the results from the household survey, another round of explanatory IDIs and FGDs were performed.

### Study Area

This study was conducted in the Kintampo North and South Districts located within the forest-savannah transitional ecological zone in the Bono East Region of Ghana. It covers an area of 7,162 km^2^ with a resident population of approximately 156,145 as at 2016 (20). The study setting is largely rural and subsistence farming is the major occupation. As a common practice, inhabitants in this study area initiate treatment for diseases at home, then continue to the Licensed Chemical Sellers Shops (over-the-counter) or pharmacies, and finally to health facilities if the illnesses do not resolve (21). There are two public hospitals, 12 health centers/ clinics, and about 30 Community-based Health Planning and Services (CHPS) compounds in the area. For the private health facilities, there are four clinics, two maternity homes, four pharmacies, and 86 Licensed Chemical Shops (over-the-counter medicine sellers). These facilities provide health services to urban as well as to the majority of deprived rural community members in the study area.

### Data Collection

#### Quantitative Data

##### Determinants of inappropriate antibiotic use

Following the qualitative interviews and using the results reported in a previous paper (19) to refine the questionnaire, a longitudinal household survey was conducted to examine potential factors which influence antibiotic use at the household level. The data collection focused on antibiotic use in the past month. The questionnaire explored the healthcare characteristics of respondents including how the cost of healthcare was covered as well as the source of antibiotics obtained. The household and socio-demographic characteristics including socioeconomic status, place of residence, age, sex, level of education, occupation, and marital status were extracted from the Kintampo Health and Demographic Surveillance System (KHDSS) database (22). The KHDSS, which is managed by the Kintampo Health Research Center (KHRC), collects and routinely updates the health and demographic information of residents, and helps in selecting study participants and following them up in the community in the course of studies. The database is updated yearly and was updated prior to this survey. The household socioeconomic status was based on the type of assets including telephone, refrigerator, solar panel, generator, radio, television, video deck, multi-TV, bicycle, motorcycle, vehicle Kiosk/Store, PC/laptop available in the participant's household. Other variables included home ownership, type of building material, source of drinking water, type of toilet facility, availability of electricity and household ownership of domestic animals such as poultry, goats, sheep, pigs, cattle, rabbits, and snails.

##### Selection of households

A total of 1,100 households was randomly selected from the KHDSS database which contains a list of all households in the study area (22).

##### Data collection procedures

Selected households were visited by trained field staff at two time points: 6 months apart within a year. The households were visited by the same field staff over the study period. The head of selected households or their representative provided consent for the survey. Survey questions for households were read aloud, and the household representative's answers were recorded by the field staff. The field staff inspected the household medicine box/bag for the presence of antibiotics when permitted and reported his/her findings back to the household representative. The field staff then enquired if any of the household members had used antibiotics in the last 1 month. When a respondent was identified to have taken antibiotics within the past month, a questionnaire was completed on antibiotic use by the user after seeking consent. A survey in the households took about 45 min for the first visit. A survey for the second visit took about 10 min because only the questions about recent antibiotic use were repeated.

##### Sample size

The sample size used for this study was 1,100 household. The detailed description and calculation of the sample size for this study has been reported in the published study protocol (18). In brief, from previous studies, it was expected that on average about 70% of antibiotic use will be classified as inappropriate. With an objective of studying the determinants of inappropriate use of antibiotics, a logistic regression approach was proposed, which resulted in a sample size of at least 1,000 households to participate in two consecutive surveys. To avoid the need for subsequently replacing households that may decline participation, 1,100 households were recruited and all were duly followed up.

##### Data management and analysis

The main study outcome was inappropriate antibiotic use, which was defined as either antibiotic use without prescription, not completing treatment course or non-adherence to instruction for antibiotic use from the provider (formal or informal) (18). ‘Yes’ responses to all or any of the three questions by a respondent was considered to be inappropriate antibiotic use. Antibiotic use episodes identified in the two surveys over the 1 year study period were combined for analysis. Data were analyzed using STATA version 14.0 (Stata-Corp, College Station, TX). An overall wealth index was computed for households using principal component analysis (PCA) as described in Vyas and Kumaranayake (23). Using the entire KHDSS population as the reference, study households were grouped by their wealth indices into low and high socio-economic status (SES). The first 60% of study households were classified as households with low SES and the remaining 40% as households with high SES. A mixed effect logistic regression analysis (multilevel analysis) was used to assess the association between inappropriate antibiotic use and household and socio-demographic, and healthcare characteristics of antibiotic users. Considering the repeated data collected on antibiotic use among households and individuals, we allowed for random effects due to households and individuals within households. The fixed-effects parameters were reported as odds ratio with their 95% confidence interval. A univariate mixed effects analysis was first conducted to assess the independent effect of each of the covariates. At a level of 10%, significant variables from the univariate analysis were included in a multivariate mixed effects analysis. With the multivariate mixed effects analysis, significance was established at 5%. With regards to the effect of socio demographic characteristics on inappropriate antibiotic use, we were interested in age, sex, level of education, occupation, and marital status. However, literature suggests that socioeconomic status and level of education tend to be strongly correlated (24). An assessment of this in our data showed a significant correlation (*p* < 0.001) between socioeconomic status and level of education. Hence, level of education was not included in the multivariate analysis. Descriptive statistics with frequencies and percentages were used to describe the factors which determine the types of drug suppliers respondents attend. Specifically respondents were asked to select the reason why they first visit the types of drug suppliers they attend when they are mildly ill.

#### Qualitative Data

To further explain the results from the household survey, another round of IDIs and FGDs was performed to discuss factors which influence inappropriate antibiotic use.

##### Selection of FGD and IDI participants

Two FGDs -male group (6 respondents) and female group (8 respondents)—were conducted among adult community members. We aimed to include 6–8 community members in each group. Field staff of KHRC who work in the study area contacted community members and explained the purpose of the study to them. Respondents who were 18 years or older and were willing to participate were included in the study without taking their socioeconomic status into consideration. FGD respondents included five farmers, six traders, two hairdressers and one was unemployed. Four IDIs were conducted among suppliers of antibiotics including one pharmacists, one dispensing technician and two Licensed Chemical Sellers (LCS)/Over-the-counter medicine sellers (OTC). IDI respondents were purposely selected and they largely represent the types of antibiotic supplier/dispensers (including public and private) in the study area which were previously identified and mapped (19). Antibiotic suppliers participated in the IDIs if they were 18 years or older and dispense or sell antibiotics.

##### Data collection procedures

The purpose and procedures of the study were explained to participants. FGDs were held in Twi dialect (a widely spoken local dialect) under a tree or a shed. Antibiotic suppliers IDIs were conducted in Twi or English in the premises where the medicines were sold or dispensed to clients at a time when attendance was very low to avoid interruption of their business. FGD and IDI sessions were audio recorded and were facilitated by a trained moderator and a note taker. IDIs and FGDs lasted for about 20 min and 1 hour, respectively. Interview sessions were brought to an end when the moderator had exhausted all questions and emerging issues.

##### Data management and analysis

Qualitative data were managed and analyzed thematically. The audio recordings of the interviews conducted in English and Twi were translated and transcribed into English verbatim by the researchers. Transcripts were reviewed to match the audio recordings and once verified they were imported into NVivo 10. After this, *a priori* themes were drawn from sociodemographic, healthcare characteristics and aspects of inappropriate antibiotic use to guide the coding of transcripts. Results were presented as narrative text in order to support quantitative findings (19).

##### Ethical issues

Ethics approvals for the conduct of this study were received from the Oxford University Tropical Research Ethics Committee (OxTREC, Reference: 31–15), Kintampo Health Research Center (KHRC) Ethics Review Committee (FWA 00011103 / IRB Registration 0004854), and Ghana Health Service Ethics Review Committee (FWA 00020025 / IRB Registration 0007714). Written informed consent was sought from all respondents after the aim and objectives of the study had been explained. Illiterate participants provided their consent through a thumb-print, which was endorsed by the signature of a literate witness who was not a member of the study staff. In addition to the signed/thumb-printed informed consent forms that were kept by the research team, prospective participants were also given a copy bearing their signature/thumb-print and the signature of the researcher or a designated person. Anonymity and confidentiality of identity and information provided by respondents were assured.

## Results

### Description of Households

A total of 1,100 households was included in the survey on antibiotic use out of which 705 (64.1%) had low socio-economic status and 675 (61.4%) lived in rural communities. Of the 1,100 households, 585 (53.2%) had used antibiotics in the 1 month prior to the survey (any person in household taken an antibiotic). Out of the 585 households which used antibiotics, 364 (62.2%) were of low socioeconomic status and majority, 380 (65.0%), of the households were in rural communities. For the non-antibiotic use households, 341 (66.2%) were of low socioeconomic status and, 295 (57.3%), of the households were in rural communities (Table 1).

**Table 1:** Description of study households.

	**All households (*****N*** **=** **1100)**		**Antibiotic use households (*****N*** **=** **585)**		**Non-antibiotic use households (*****N*** **=** **515)**	
**Household characteristics**	**Frequency**	**Percentage**	**Frequency**	**Percentage**	**Frequency**	**Percentage**
**Socioeconomic status**						
Low	705	64.1	364	62.2	341	66.2
High	350	31.8	203	34.7	147	28.5
Missing	45	4.1	18	3.1	27	5.2
**Place of residence**						
Rural community	675	61.4	380	65.0	295	57.3
Urban community	425	38.6	205	35.0	220	42.7

### Description of Antibiotic Use and Antibiotic Users

**Figure 1:**
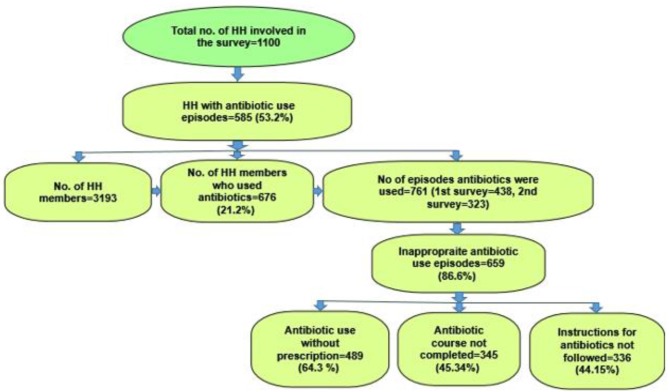
Household (HH) survey and antibiotic use.

Out of 585 households, 437 households used antibiotics in the first survey, 322 households used antibiotic in the second survey and 174 households used antibiotics in both surveys. In these 585 antibiotic use households, there were 3,193 household members of whom 676 (21.2%) had used antibiotics for 761 episodes of illness on an individual level. Out of the 761 antibiotic use episodes, 659 (86.6%) were used inappropriately. In all the inappropriate antibiotic use episodes, 489 (64.3%) were used without prescription and 345 (45.3%) treatment courses were not completed. Also, instruction for use were not followed in 336 (44.15%) antibiotic use episodes (Figure 1). Of the 676 antibiotic users, 232 (34.3%) were between the ages of 21–40 years, 433 (64.0%) were females and 250 (37.0%) had no education. Also, 456 (67.5%) were unemployed and 364 (53.8%) were not married (Table 2).

**Table 2:** Demographic characteristic of antibiotic users.

**Demographic characteristic of antibiotic users**	**Frequency (*N* = 676)**	**Percentage**
**Age**		
20 and below	157	23.2
21–40	232	34.3
41–60	195	28.9
60 and above	92	13.6
**Sex**		
Male	243	36.0
Female	433	64.0
**Level of education**		
None	250	37.0
Primary school	153	22.6
Middle/Junior high school	120	17.8
Secondary school+	153	22.6
**Occupation**		
Employed	456	67.5
Unemployed	220	32.5
**Marital status**		
Married	364	53.8
Not married	312	46.2

### Household, Socio-Demographic, Healthcare Characteristics and Inappropriate Antibiotic Use

In the univariate mixed effects logistic regression analysis, socioeconomic status (Odds Ratio (OR): 0.5, 95% CI: 0.28–0.86, *p*-value: 0.014), place of residence (OR: 0.6, 95% CI: 0.3–0.9, *p*-value: 0.027), level of education (OR: 0.4, 95% CI: 0.2–0.7, *p*-value: 0.001) and paying for healthcare through national health insurance (OR: 0.4, 95% CI: 0.2–0.7, *p*-value: 0.006) were significantly associated with inappropriate antibiotic use. Also, seeking for healthcare at the health center (OR: 2.3, 95% CI: 1.1–4.7, *p*-value: 0.028), pharmacy (OR: 4.6, 95% CI: 1.6–13.2, *p*-value: 0.005) and with drug peddlers (OR: 0.3, 95% CI: 0.1–0.8, *p*-value: 0.013) were significantly associated with inappropriate antibiotic use.

However, in the multivariate mixed effects logistic regression analysis, socioeconomic status (OR: 0.7, 95% CI: 0.40–1.07, *p*-value: 0.094) and place of residence (OR: 0.7, 95% CI: 38–1.56, *p*-value: 0.220) were not significantly associated with inappropriate antibiotic use. Seeking for healthcare from the drug peddlers (OR: 0.5, 95% CI: 0.2–1.1, *p*-value: 0.073) was marginally associated with inappropriate antibiotic use. Respondents who do not pay for healthcare with the national health insurance (OR: 2.9, 95% CI: 1.1–7.4, *p*-value: 0.026) were more likely to use antibiotics inappropriately compared to users of national health insurance. Also, compared to those who seek healthcare at the health center, respondents who do not seek healthcare at the health center (OR: 2.4, 95% CI: 1.2–5.0, *p*-value: 0.018) were more likely to use antibiotics inappropriately. Lastly, compared to those who seek healthcare at the pharmacy, respondents who do not seek healthcare at pharmacy (OR: 4.6, 95% CI: 1.7–13.0, *p*-value: 0.003) were more likely to use antibiotics inappropriately (Table 3).

**Table 3:** The association between household, socio-demographic characteristics of antibiotic users and inappropriate antibiotic use.

		**Antibiotic use episodes** ***n*** **(%)** ***N*** **=** **761**		**Univariate mixed effects logistic regression Odds Ratio (90% CI)**	***P*-Value**	**Multivariate mixed effects logistic regression Odds Ratio (95% CI)**	***P*-Value**
		**Appropriate (*N* = 102)**	**Inappropriate (*N* = 659)**				
**HOUSEHOLD AND SOCIO-DEMOGRAPHIC CHARACTERISTICS ANTIBIOTIC USERS**							
**Socioeconomic status**							
Low		51 (10.69)	426 (89.3)	Reference group (REF)		REF	
High		47 (17.87)	261 (82.1)	0.5 (0.3–0.9)	0.014	0.7 (0.4–1.1)	0.094
**Place of residence**							
Live in rural community		56 (11.2)	443 (88.8)	REF		REF	
Live in urban community		46 (17.6)	216 (82.4)	0.6 (0.3–0.9)	0.027	0.7 (0.4–1.6)	0.220
**Age**							
20 and below		21 (12.7)	144 (87.3)	REF			
21–40		37 (14.3)	222 (85.7)	0.9 (1.4–1.7)	0.644		
41–60		32 (13.9)	198 (86.1)	0.9 (0.4–1.8)	0.737		
60 and above		12 (11.2)	95 (88.8)	1.1 (0.5–2.7)	0.782		
**Sex**							
Male		35 (12.5)	245 (87.5)	REF			
Female		67 (13.9)	414 (86.1)	0.9 (0.5–1.5)	0.616		
**Educational level**							
None		27 (9.6)	256 (90.4)	REF			
Primary school		19 (10.8)	157 (89.2)	0.9 (0.4–1.7)	0.666		
Middle/Junior high school		22 (15.8)	116 (84.2)	0.6 (0.3–1.1)	0.075		
Senior high school+		34 (20.7)	130 (79.3)	0.4 (0.2–0.7)	0.001		
**Occupation**							
Employed		66 (12.7)	455 (87.3)	REF			
Unemployed		36 (15.0)	204 (85.0)	0.8 (0.5–1.4)	0.417		
**Marital status**							
Married		52 (12.6)	360 (87.4)	REF			
Not married		50 (14.4)	299 (85.7)	0.9 (0.6–1.3)	0.520		
**HEALTHCARE CHARACTERISTICS OF ANTIBIOTIC USERS**							
**How the costs for healthcare is covered**							
National health insurance	Yes	96 (15.1)	541 (84.9)	REF		REF	
	No	6 (4.8)	118 (95.2)	4.1 (1.5–11.0)	0.006	2.9 (1.1–7.4)	0.026
**Types of drug supplier respondents attend**							
District hospital	Yes	79 (13.4)	511 (86.6)	REF			
	No	23 (13.4)	148 (86.6)	1.0 (0.5–1.8)	0.993		
Health center	Yes	20 (38.3)	73 (78.5)	REF		REF	
	No	82 (21.3)	586 (87.7)	2.3 (1.1–4.7)	0.028	2.4 (1.2–5.0)	0.018
Community-based Health Planning and Services	Yes	5 (9.4)	48 (90.6)	REF			
	No	97 (13.7)	611 (86.3)	0.6 (0.2–1.9)	0.414		
Private Clinic	Yes	29 (14.7)	169 (85.4)	REF			
	No	73 (13.0)	490 (87.0)	1.2 (0.7–2.1)	0.529		
Pharmacy	Yes	10 (35.7)	18 (64.3)	REF		REF	
	No	92 (12.6)	641 (87.5)	4.6 (1.6–13.2)	0.005	4.6 (1.7–13.0)	0.003
Licensed Chemical Sellers	Yes	58 (12.3)	414 (87.7)	REF			
	No	44 (15.2)	245 (84.8)	0.7 (0.4–1.3)	0.270		
Drug peddlers	Yes	8 (6.1)	123 (93.9)	REF		REF	
	No	94 (14.9)	536 (85.1)	0.3 (0.1–0.8)	0.013	0.5 (0.2–1.1)	0.073

### Factors That Determine the Types of Drug Suppliers' Respondents Attend

Distance to health facility 445 (65.83%), trust for medicine supplier 552 (77.22%) and disease severity 519 (76.8%) were important factors which influence the type of drug suppliers' respondents go to for healthcare (Table 4).

**Table 4:** Description of factors that are associated with the types of drug suppliers respondents attend.

**Factors**	**Frequency (*N* = 676 antibiotic users)**	**Percent**
Distance to facility		
Yes	445	65.8
No	231	34.2
Cost of medicine		
Yes	218	32.3
No	458	67.8
Trust in medicine supplier		
Yes	522	77.2
No	154	22.8
Popularity of facility		
Yes	207	30.6
No	469	69.4
Disease severity		
Yes	519	76.8
No	157	23.3
Age of sick person		
Yes	78	11.5
No	598	88.5

### Qualitative Themes Associated With Inappropriate Antibiotic Use

FGDs and IDIs were conducted to further explore how household, sociodemographic, health care characteristics and the type of health provider respondents attend influence inappropriate antibiotic use. Emerging themes reported include how cost of medicines and distance to health facilities seem to facilitate inappropriate antibiotic use.

It emerged that relative high cost of medicines including antibiotics influence inappropriate antibiotic use. Contrary to findings from the quantitative study (socioeconomic status was not significantly associated with inappropriate antibiotic use), antibiotic users with low socioeconomic status have a higher tendency to use antibiotics inappropriately. It was revealed that clients with low socioeconomic status tend to buy fewer antibiotics than expected with the understanding that, they will come back and buy the remaining antibiotics when they have money available. The excerpt below demonstrates how cost of antibiotics contributes to inappropriate antibiotic use.

…*at the drug stores (licensed chemical sellers) they try as much as possible to know the amount of money you have on you and then prescribe medicines according to money (FGD with women respondent#3)*

*Sometimes it's about the money. The person cannot afford the full dose….if someone has to take 21 capsules of amoxicillin which cost three Ghana cedis (USD 0.55) and the person has only one Ghana cedi (USD 0.18), what do we do? We can't also let the person go home without any treatment so we sell to them*. (IDI with dispensing technician at a pharmacy)

A Licensed chemical seller also indicated that people regularly buy antibiotics in installments due to cost. The respondent further explained that the real challenge is that clients are less likely to come back and buy the rest of the medicines once they get better.

*The clients think that buying antibiotics in installments is a normal practice. Some will say give it (antibiotics) to me and when it is about getting finish I will come and purchase the rest but once he/she takes it (antibiotics) and sees that the symptoms are reducing they (clients) will forget about the rest*. (IDI with a Licensed Chemical Seller)

However, dispensers at pharmacy shops alluded that they are sometimes not comfortable selling incomplete doses of antibiotics to clients, but that if they do not, the clients are likely to buy the medicines from drug peddlers.

…*we cannot say that because of regulation if the person comes and cannot afford the full course don't sell it; people will stay in their houses and buy from drug peddlers*. (IDI with dispensing technician at a pharmacy)

Further findings from the qualitative study give credence to how long distances to approved sources of antibiotics facilitates the activities of untrained and unlicensed drug peddlers, whose dispensing activities could possibly influence inappropriate antibiotic use.

*The doctor who can tell you about the medicine which will help you; sometimes the sickness is not severe so we go to the drug shop (licensed chemical seller) because the distance to go and see the doctor is far*. (FGD with Men respondent#4)

*I went to my farm in a village which is 150 kilometers away from the district capital. A guy was weeding and had a cutlass wound and was bleeding profusely; we didn't have anything like first aid so we went to him (drug peddler). He had some spirit and did some basic treatment*. *He doesn't have a license so what he is doing is against the law but in this instance he saved a life*. (IDI with dispensing technician at a pharmacy)

The dispenser further suggested that these drug peddlers should be trained to do the right thing [dispense medicines and antibiotics appropriately] considering that they provide healthcare in very remote communities where there are no public or private health facilities and trained dispensers are not prepared to go there.

*These guys [drug peddlers] put drugs on motor bikes and travel to rural communities where nobody will even go and sell anything and no trained dispenser will go and stay there. So the only option to me is for the government to select some of the peddlers and devise a way to train them so that they will do the right thing*. (IDI with dispensing technician at a pharmacy).

## Discussion

This paper examined factors which influence inappropriate antibiotic use at the community level in rural Ghana. Overall, inappropriate antibiotic use was high (86.6%) and mainly influenced by out-of-pocket payment for healthcare, seeking for healthcare outside health centers and pharmacies. Also, the relatively high cost of medicines including antibiotics facilitates inappropriate antibiotic use. Gaining insight into these factors will contribute to the development of context-specific and effective policies and interventions to optimize antibiotic use and curb resistance.

In our study context inappropriate antibiotic use appears to be the norm due to easy access to antibiotics over-the-counter with or without prescription (19, 25). These findings on high inappropriate antibiotic use are comparable to findings from other study (26) and contribute to the loss of first line antibiotics as effective treatment (27). Similar to other LMIC, over 50% of antibiotics are purchased and used over-the-counter and from unapproved sources which largely contributes to inappropriate antibiotic use (28, 29). However, it is important to note that the number of antibiotic use episodes reduced from 438 in the first survey to 323 in the second survey. This is possibly due to the prescribing/dispensing patterns in the study area (30). The first survey was mainly carried out in the first quarter of the year during which respiratory tract infections are generally high due to change in weather as a results of the harmattan (dry season with lot of dust) compared to the last half of the year. These diseases require the use of antibiotics which may be obtained with or without prescription.

Antibiotic users who do not pay for healthcare through the NHIS were more likely to use antibiotics inappropriately. As indicated earlier, NHIS registered members receive healthcare for some common diseases without paying upfront for medicines and services provided (11). This largely encourages people to seek for healthcare in hospitals and other accredited sources (11). Antibiotic users who have not registered for the NHIS were therefore largely inclined to buy antibiotics from LCS/OTC and drug peddlers who could sell incomplete courses to them if they cannot afford the full course and this predisposes them to inappropriate antibiotic use (19, 31). This finding could further be explained in the context of socioeconomic status as a determinant of inappropriate antibiotic use (32, 33). For instance, out of the 40% of the people who have registered for NHIS in Kintampo north municipality as at 2016, only 24.9% have renewed their annual premiums with the remaining (about 75%) citing high cost of premium (GHS30.00; $5.43 per year) as the main barrier (34, 35). Distance to and cost of travel to NHIS offices, and delays were also found to be barriers to NHIS registration (34, 36).

The qualitative study further highlights the relative high cost of antibiotics as a determinant of inappropriate use. This emphasizes the importance of cost of medicines as a determinant of community antibiotic use and practices (19, 31, 37). High costs of medicine have been associated with inappropriate antibiotic use in other studies (32, 33, 38). In our study context where self-medication is common, community members opt to purchase medicines including antibiotics from LCS/OTC and drug peddlers because medicines could sometimes be obtained on credit. As indicated earlier, these medicine sellers who are largely influenced by financial gains sometimes dispense less antibiotics than required when clients cannot afford a full course leading to inappropriate use (19, 31).

Our study further revealed that antibiotic users who do not obtain antibiotics at health centers or pharmacies were more likely to use antibiotics inappropriately. As indicated earlier, such antibiotic users were predisposed to buy antibiotics from unapproved sources such as LCS/OTC which could result in inappropriate use. This was further substantiated by our findings where seeking healthcare from drug peddlers was marginally associated with inappropriate use. In Ghana, pharmacies are licensed to dispense antibiotics under recommendation and supervision of a practicing pharmacist likewise health centers which are managed by physician assistants (39, 40). Pharmacies and health centers are therefore expected to provide adequate services including appropriate provision of some categories of antibiotics.

Distance to health facilities as a possible determinant of the source of antibiotics could result in inappropriate antibiotic use. Rural residents who are generally of low socioeconomic status often buy antibiotics from unapproved sources such as LCS/OTC and drug peddlers for reasons such as distance to health facilities and pharmacies (7, 19). In Ghana, health facilities which are approved to dispense antibiotics are mainly located in the district capitals which are about 5–20km from rural residents (41). This is inconsistent with WHO standard which indicates 5 km radius access to health care for every person (42, 43). Half of the population cannot consult a medical officer within 5 km in Ghana (42). Similarly, a study in India found that lack of access to approved sources of antibiotics and travel costs influence acquisition of antibiotics from unapproved dispensers in their communities (37).

Household socioeconomic status and place of residence were independently associated with inappropriate antibiotic use. However, after adjusting for other variables including “how healthcare costs are covered” and “type of drug supplier antibiotic users attend,” household socioeconomic status and place of residence were not significantly associated with inappropriate antibiotic use. The social structure of households in our study context is largely homogenous (44) hence the probable absence of differential significant association in household characteristics and inappropriate antibiotic use.

## Strengths

To the best of our knowledge this is the first study of its kind in Ghana which used mixed methods and a large sample size to investigate contextual factors which influence inappropriate antibiotic use. Findings from this study will contribute to the baseline data and surveillance activities of the Ghana National AMR platform which is implementing various strategies to combat antibiotic resistance. This study can therefore provide useful insights for local practice and public health policy.

## Limitations

The measurement of inappropriate antibiotic use was based on the responses from respondents. This could possibly lead to some socially desirable responses considering that some respondents may assume they could be perceived to be doing the “wrong thing” if they do not give affirmative responses. However, their responses provided possible pointers or clues to how appropriate or inappropriate antibiotics were used at the community level or outside healthcare settings for possible future interventions to improve use and curb resistance.

## Recommendations

There is a need for ministries of health and healthcare agencies in Ghana and LMIC to enhance access to approved health services and health care insurance, and to provide affordable antibiotics to improve use at community level especially in rural settings. Expanding the subscription for national health insurance and paying claims to accredited health facilities without delays is therefore key to enhancing access and appropriate antibiotic use. In addition to expanding health insurance subscription, the ministry of health and relevant agencies should intensify public awareness campaign about the harmful effects of antibiotic resistance and improving the standard of living of citizen and access to good healthcare. Furthermore, the ministry of health should also consider training, recruiting and providing incentives to pharmacist to establish pharmacies in rural communities to help reduce the OTC sales of antibiotics. Meanwhile, the ministry of health and other relevant regulatory agencies (the pharmacy council in the case of Ghana) should consider training LCS/OCT and equipping CHPS facilities to dispense some categories of antibiotics. LCS/OTC and CHPS are close to community members and will greatly improve access and use.

## Conclusion

Inappropriate antibiotic use is common and influenced by healthcare characteristics. These include out-of-pocket payment for healthcare or paying for healthcare without the national health insurance and seeking for healthcare outside health centers or pharmacies. Other interrelated determinants of inappropriate use were relative high cost of medicine including antibiotics and distance to health facilities. Considering that inappropriate use is high and appears to be the norm in this study, well enforced policies are needed to change the status quo.

## Data Availability Statement

The datasets generated for this study are available on reasonable request to the corresponding author.

## Ethics Statement

Ethics approvals for the conduct of this study were received from the Oxford University Tropical Research Ethics Committee (OxTREC, Reference: 31-15), Kintampo Health Research Center (KHRC) Ethics Review Committee (FWA 00011103/IRB Registration 0004854) and Ghana Health Service Ethics Review Committee (FWA 00020025/IRB Registration 0007714). The patients/participants provided their written informed consent to participate in this study.

## Author's Note

This paper is part of a Ph.D. study and HW is the Principal supervisor for the Ph.D. and a co-author on this paper. He is also one of the editors hosting the section of this journal.

## Author Contributions

SA-A, HW, and KA contributed to study conception and design. Data collection was carried out by SA-A, EB-K, and MA. Data management was done by OA. Data analysis was conducted by SA-A, FO, AT, HW, KA, MH, SG, and JK. All authors contributed to interpretation of findings and reviewed the manuscript.

### Conflict of Interest

The authors declare that the research was conducted in the absence of any commercial or financial relationships that could be construed as a potential conflict of interest.
